# Evaluation of the Impact of Mandating Health Care Providers to Offer Hepatitis C Virus Screening to All Persons Born During 1945–1965 — New York, 2014

**DOI:** 10.15585/mmwr.mm6638a3

**Published:** 2017-09-29

**Authors:** Colleen A. Flanigan, Shu-Yin J. Leung, Kirsten A. Rowe, Wendy K. Levey, Andrea King, Jamie N. Sommer, Johanne E. Morne, Howard A. Zucker

**Affiliations:** ^1^Bureau of Hepatitis Health Care, AIDS Institute, New York State Department of Health; ^2^Office of Program Evaluation and Research, AIDS Institute, New York State Department of Health; ^3^Medicaid Finance Systems and Research, AIDS Institute, New York State Department of Health; ^4^Bureau of Communicable Disease Control, New York City Department of Health and Mental Hygiene; ^5^Bureau of Communicable Disease Control, New York State Department of Health; ^6^Office of the Director, AIDS Institute, New York State Department of Health; ^7^Office of the Commissioner, New York State Department of Health.

Approximately 75% of all hepatitis C virus (HCV) infections in the United States and 73% of HCV-associated mortality occur in persons born during 1945–1965, placing this birth cohort at increased risk for liver cancer and other HCV-related liver disease (*1*). In the United States, an estimated 2.7 million persons are living with HCV infection, and it is estimated that up to 75% of these persons do not know their status. Since 2012, CDC has recommended that persons born during 1945–1965 receive one-time HCV testing. To increase the number of persons tested for HCV and to ensure timely diagnosis and linkage to care, in 2014, New York enacted a hepatitis C testing law that requires health care providers to offer HCV antibody screening to all persons born during 1945–1965 who are receiving services in primary care settings or as hospital inpatients, and to refer persons with positive HCV antibody tests for follow-up health care, including an HCV diagnostic test (i.e., HCV RNA).* The New York State Department of Health (NYSDOH) used survey data from clinical laboratories and Medicaid claims and encounter data, and state and New York City (NYC) HCV surveillance data to assess the number of persons tested for HCV and number of persons with newly diagnosed HCV infections who were linked to care. During the first year of the HCV law implementation, there was a 51% increase in specimens submitted for HCV testing to surveyed clinical laboratories; testing rates among active Medicaid clients increased 52%, and linkage to care among persons with newly diagnosed HCV infection increased approximately 40% in New York and 11% in NYC. These findings highlight the potential for state laws to promote HCV testing and the utility of HCV surveillance and Medicaid claims data to monitor the quality of HCV testing and linkage to care for HCV-infected persons.

Before the law's effective date (January 1, 2014), NYSDOH conducted activities to inform providers of the new law, including issuing a provider letter, conducting regional stakeholder meetings and a statewide webinar, and hosting briefings with existing councils and task forces. A frequently asked questions (FAQ) document was also developed and disseminated widely.

To assess the number of persons screened for HCV infection before and after implementation of the HCV testing law in 2014, a survey of clinical laboratories was conducted. Monthly counts of specimens collected from January 2013 through December 2014 for HCV testing from persons born during 1945–1965 were requested from 163 laboratories holding NYSDOH Clinical Laboratory Evaluation Program permits for HCV testing. Twelve (7.4%) laboratories did not meet eligibility requirements^†^ and were excluded from the evaluation.

In addition to the laboratory survey, New York Medicaid data were used to assess trends in HCV testing before and after implementation of the law. Deidentified Medicaid claims and encounter data were used to create monthly denominators for the entire Medicaid population over a 3-year period (January 2012 through December 2014). Only Medicaid recipients born between 1945 and 1965 (aged 50–70 years in 2015) receiving paid services during a given month during the study period (active Medicaid clients) were included in this analysis. Rates of HCV testing per 1,000 Medicaid recipients were calculated based on the number of persons for whom HCV testing procedure codes (current procedural terminology [CPT]) were billed.^§^ Medicaid recipient records were deduplicated within each month to reflect only one test per person per month. Records were not deduplicated across months.

To assess linkage to care after implementation of the law, NYSDOH and NYC Department of Health and Mental Hygiene HCV surveillance data were reviewed. The proportions of nonincarcerated persons born during 1945–1965 with newly diagnosed cases of confirmed HCV infection (*2*) who were linked to care during the preenactment period (January 2011–December 2013) and the postenactment period (January 2014–December 2014) were compared. Linkage to care was defined as documentation of either 1) two or more positive HCV RNA tests (excluding reflex RNA testing) or 2) one positive HCV RNA test (excluding reflex RNA testing) and an HCV genotype test within 6 months of the initial positive HCV antibody result. In reflex RNA testing, a positive antibody test result triggers an automatic RNA test by the laboratory on the same specimen. For this analysis, reflex testing was defined as an HCV RNA test with the same collection date as the HCV antibody test. The rationale for excluding reflex testing is that a reflex RNA test is automatic and does not necessarily indicate an engagement in care. These laboratory data were made available through the Electronic Clinical Laboratory Reporting System. Linkage to care was assessed among active Medicaid clients receiving Medicaid services statewide. Among Medicaid recipients receiving HCV antibody testing, those who also received RNA testing^¶^ during the same year they were initially tested were considered linked to care; because Medicaid claims data do not distinguish reflex testing, these results might include reflex testing.

Among the 151 laboratories eligible for the survey, 116 (76.8%) responded, 106 (91.4%) of which provided 24 months of usable data for analysis. Among laboratories that provided 24 months of data, the monthly rates of increase for 2013 (preenactment) and 2014 (postenactment) were assessed by fitting two linear trend lines to the 2013 and 2014 monthly data, respectively.

Data from the 106 responding laboratories that provided 24 months of data indicated a 51.1% increase in the number of specimens collected for HCV testing from persons born during 1945–1965, from 538,229 in 2013 to 813,492 in 2014 (Figure 1). During 2013, the average rate of increase was approximately 404 specimens per month. In 2014, the average rate of increase was 1,091 specimens per month.

**FIGURE 1:**
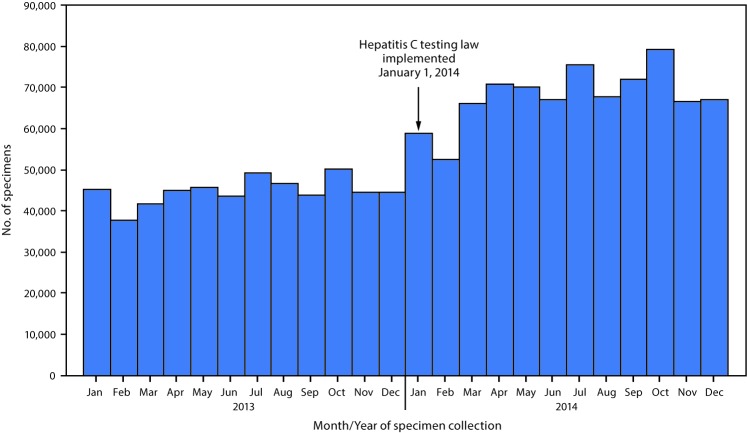
Number of specimens collected for hepatitis C virus testing from persons born during 1945–1965 by participating clinical laboratories holding New York Clinical Laboratory Evaluation Program permits (N = 106) — New York, January 2013–December 2014

New York Medicaid data from 2012 to 2014 also demonstrated an increase in HCV testing. Before the law was enacted, the average monthly HCV testing rate for persons born during 1945–1965 was 8.4 per 1,000 active Medicaid clients in 2012 and 8.8 in 2013. After enactment of the law in January 2014, the average monthly HCV testing rate rose to 12.8 per 1,000, representing a 52% increase in the average monthly testing rate from 2012 to 2014 (Figure 2). In contrast, the monthly rate of HCV testing increased only slightly among active Medicaid clients born before 1945 or after 1965, from 4.5 per 1,000 active clients during 2012 to 4.8 in 2013 and 5.6 in 2014, an overall 24% increase in the average monthly testing rate.

**FIGURE 2:**
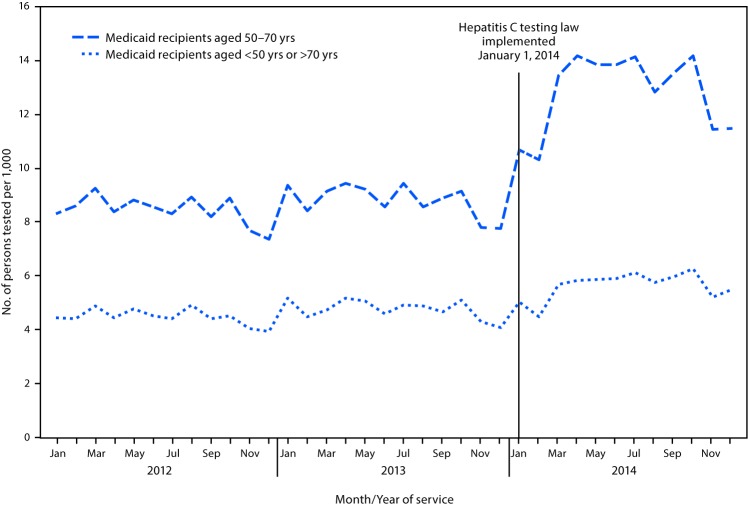
Rate of hepatitis C virus testing* per 1,000 Medicaid recipients, by age cohort — New York, 2012–2014 * Procedure codes 86803 and 86804.

Analysis of HCV surveillance data indicated a 39.8% increase (from 24.1% to 33.7%) in the percentage of persons with newly diagnosed HCV infection who were linked to care in New York, and an 11.2% increase (from 19.5% to 21.7%) in NYC during 2014 (after enactment of the law) compared with 2011–2013. Medicaid data indicated an overall rate increase of 35% from 13,839 to 18,614 between 2013 and 2014.

## Discussion

Implementation of the New York law mandating health care providers to offer HCV testing to persons born during 1945–1965 was associated with an increase in HCV testing, and an increase in the percentage of persons with newly diagnosed HCV infections who were linked to care. Marked increases in the number of HCV tests performed and rates of testing were observed immediately after enactment of the law and remained steady over a 12-month period. Smaller increases were noted in the number of persons who accessed care after receiving a positive HCV screening test result.

The use of multiple and complementary data sources in the evaluation was necessary to document the changes in HCV screening and linkage to care since enactment of the law. For instance, determining the extent of HCV screening and linkage to care in New York was not possible through examination of surveillance data alone because reporting of negative test results was not required at the time of the evaluation. The use of laboratory survey data provided a simple and direct way to assess the relative changes in the number of HCV screening specimens tested over a 24-month period. The use of Medicaid claim and encounter data complemented the findings from the laboratory survey by allowing a comparison of HCV testing rates among persons born during 1945–1965 with rates among persons born before 1945 and after 1965. The sharp rise in the testing rate among New York active Medicaid clients aged 50–70 years after implementation of the testing law contrasted with the more gradual increase in HCV screening rates among younger and older Medicaid clients for whom the law does not apply.

The findings in this report are subject to at least five limitations. First, the use of observational data did not allow for controlling for all the possible factors that might have contributed to the observed increases. For example, the reasons for the gradual increase in HCV testing in the year before the law was implemented that were identified by the laboratory survey are not known, but the gradual increase might have been affected by educational efforts around the recommendation for screening of persons in this age group and increased awareness of the CDC recommendation, and an increase in general HCV awareness. Second, although response rates from the participating laboratories were high (77%), the findings are based on only those laboratories that responded to the inquiry and might not be representative of all laboratories. Third, there is no standardized or universally accepted indicator for linkage to care, and the proxies developed for the analysis of surveillance and Medicaid data have not been independently validated. Fourth, laboratories were required to report only positive HCV RNA test results during the evaluation period; therefore, persons whose HCV RNA test results were negative, but who were linked to care were not included in the analysis, possibly resulting in underascertainment. Finally, limited capacity for HCV care and treatment, especially among HCV specialists, might have negatively affected timely linkage to care. In some areas of the state, wait times for appointments can exceed 6 months. Limited resources for conducting active linkage to care might also have also negatively influenced rates.

With availability of new HCV therapies that can stop disease progression and result in a virologic cure for >90% of HCV-infected persons, testing and linkage to care for HCV-infected persons in this birth cohort are expected to reduce HCV-related morbidity and mortality and decrease deaths from liver cancer (*1*). During the first year of the law’s implementation, HCV treatments were available through the New York Medicaid Program (i.e., fee-for-service). However, prior authorization was required and disease severity restrictions were enforced.** On April 27, 2016, those disease severity restrictions were eliminated, allowing greater access to treatment. This report highlights the potential for state laws to promote HCV testing and the utility of HCV surveillance and Medicaid claims data to monitor the quality of HCV testing and linkage to care for HCV-infected persons.

**Summary**

**What is already known about this topic?**

Persons born during 1945–1965 account for approximately 75% of all hepatitis C virus (HCV) infections in the United States and 73% of HCV-associated mortality. Most infected persons do not know their status. In January 2014, New York became the first state to enact an HCV testing law, which is expected to increase the number of persons who are aware of their HCV status.

**What is added by this report?**

One year after implementation of the 2014 New York HCV Testing Law, marked increases were observed in the number of HCV screening tests and rates of testing. Increases were observed almost immediately after enactment of the law and remained steady at levels substantially higher than those in the years preceding enactment of the law. Smaller increases were noted in the number of persons who accessed HCV care following a positive HCV screening test.

**What are the implications for public health practice?**

State-level HCV testing laws could increase the number of persons who know their HCV status and of HCV-infected persons who are linked to care. With the availability of new therapies that can stop disease progression and provide a cure in most persons, testing and linkage to care for infected persons is likely to reduce HCV-related morbidity and liver cancer-associated mortality.

## Footnotes

* Required Offering of Hepatitis C Screening Testing, NY Pub Health L § 2171 (January 1, 2014). http://law.justia.com/codes/new-york/2014/pbh/article-21/title-7/2171/.

† Laboratories that only test specimens for clinical trials, end-stage renal disease, or tissue/organ donations, and laboratories that were unable to report data by birth cohort or residence, were not eligible for participation.

§ Current procedural terminology (CPT) codes 86803 or 86804 (hepatitis C antibody or hepatitis C antibody; confirmatory test (eg, immunoblot)).

¶ RNA test codes defined as follows: 87520 = infectious agent detection by nucleic acid (DNA or RNA); hepatitis C, direct probe technique; 87521 = infectious agent detection by nucleic acid (DNA or RNA); hepatitis C, reverse transcription and amplified probe technique; 87522 = infectious agent detection by nucleic acid (DNA or RNA); hepatitis C, reverse transcription and quantification.

** HCV treatment was restricted to persons with advanced liver disease (evidence of stage 3 or 4 hepatic fibrosis).
